# Reconfiguration of relationships during the process of remarriage after divorce. A qualitative study in Iran

**DOI:** 10.25122/jml-2020-0196

**Published:** 2021

**Authors:** Abbasali Yazdani, Mirtaher Mousavi, Fardin Alipour, Hassan Rafiey

**Affiliations:** 1. Department of Social Work, University of Social Welfare and Rehabilitation Sciences, Tehran, Iran; 2. Social Welfare Management Research Group, University of Social Welfare and Rehabilitation Sciences, Tehran, Iran; 3. Department of Social Work, University of Social Welfare and Rehabilitation Sciences, Tehran, Iran; 4. Department of Social Welfare Management, University of Social Welfare and Rehabilitation Sciences, Tehran, Iran

**Keywords:** rebuilt family, quality of life, Iran, remarriage, divorce

## Abstract

Marriage is highly respected and somehow sacred in eastern societies, including Iran. This qualitative research aimed to explore lived experience of remarried men and women who had experienced divorce in their relationships with their significant others. Seventeen remarried-after-divorce persons whose second marriage lasted over two years participated in the study, conducted using the content analysis method. Under the theme of inter-relationships, four categories were found, including “different spouses, different relations”, “reconfiguration of relationships with families”, “impacts on child-rearing”, and “the importance of unimportant acquaintances”. This study showed the complexity of the relationship network when another marriage happens after the first one collapsed. The other finding was that relations after divorce might not disappear completely, and relics of the first ruined shared life may strongly affect the new partnership. The influence of context-oriented issues, particularly in societies where religion and tradition are strong, was the other result. This study showed that remarriage after divorce has positive and negative consequences. Professionals, policymakers and researchers may apply the findings of the research by taking a strengths perspective.

## Introduction

Family is still predominantly defined as a social unit that originates from a formal marriage of opposite-sex couples, ideally with the function of bearing and rearing some. However, sometimes this framework is broken up by divorce. After the marriage break-up, each member of the ex-united family may take their path. Some of the ex-couples stay unmarried forever while others choose to marry again. Evidence suggests staying single after divorce may have various undesirable consequences for both genders [1, 2]. However, an unsuccessful remarriage will bring worse conditions, such as child-parent relationships, a sense of victimization, lost self-esteem, and domestic violence both for couples and children [3]. These advocate the claim of Dupuis [4] that rebuilt family is not thoroughly good or bad; thus, entering into second or more marriages must be opted very carefully. Results are different based on the cultures and places of living as well [5, 6].

In Iran, even in the urban population, the environment strongly influences the decision to remarry and the way to continue it, particularly for younger women [7]. As free liaison or having an out-of-marriage relationship between different sexes is legally forbidden and not commonly favored among the public, a significant proportion of decision to remarry instead of dating or singleness can be attributed to satisfy needs in a pro-social way. At the same time, many feel regret very soon after remarriage [8, 9]. Moreover, marriage is strongly recommended by religion and Iran's legislation, including the constitution and the civil law, which are based on Islam that encourages marriage as a value. Trying hard to keep couples married while lacking the process to strengthen infrastructures may tempt couples to have just an annoyingly superficial tie. The large number of extramarital relationships in Iran could be an indicator of dissatisfaction with marriage, even for those who remarry [10].

The authors' experiences working on divorce and newly-married couples also show that family interactions, either manipulation or support and social context, may affect the quality of life of people who remarried after divorce. However, few types of research have been conducted to investigate interfering relations in Iran. Based on the lived experiences of Iranian remarried divorcees, the present research inquired which familial or contextual relationships are important to them and how in their new shared life.

## Material and Methods

The method of content analysis was applied to find the latent meanings in qualitative data. Semi-structured interviews were used to let the participants freely express their lived experiences. This was part of a larger research with the aim of designing a questionnaire on the same topic.

### Participants

Seventeen remarried individuals – 9 men and 8 women – who experienced a divorce were included in the study. They were found via purposive sampling through various ways, including an introduction by friends, acquaintances, or other participants. Since finding the target group is not typically easy, and couples usually tend to hide their past, the researchers tried different ways to reach them. Sending inviting messages twice via social networks such as Telegram was an alternative.

Having been introduced, the first researcher (AY) had a short conversation with the participants. If they met the inclusion criteria, they were briefly briefed about the study and assured about anonymity, confidentiality, and their freedom to withdraw from the study whenever they wished. Finally, they were invited to an interview meeting that was arranged based on the participants' preferences. Inclusion criteria included participants who divorced at least once and then remarried, were in place at the time of the interview and were willing to talk about their experiences. The researchers attempted to include various participants in terms of age, education, socioeconomic status, duration of marriage and remarriage life, custody status, and others.

The participants' age ranged from 26 to 56 years (M=39.70; SD=8.34); duration of the first marriage ranged from 1 to 20 years (M=8.35; SD: 5.63); and time passed since remarriage ranged from 1 to 32 years (M=6.68; SD=7.69). One male participant had married for the third time. Eleven participants married to divorcees. Eleven cases had children from the first relationship, and seven had retained custody of the children after remarriage. Two male and two female participants started to be step-parents in remarriage and built blended families.

### Procedure

The participants were interviewed individually. To make sure they felt sufficiently comfortable and safe, the interviewees could choose to meet the researcher in their preferred place (an agreed office, public spaces, or their workplace). The second interview occurred by phone. All participants were verbally informed that confidentiality was respected and that they could stop participation at any time during the study.

After a brief introduction, the interviews began with a general statement (“shall we start with some dates like your first marriage, divorce and remarriage time?”) and gradually were guided by more exploratory questions to probe their perceived identity apprehensions. The duration of the interviews ranged from 28 to 105 minutes (M=51.8). As the research progressed, more structured questions were inferred, which were used for the next interviews.

### Data gathering

In the beginning, the data were collected by deep semi-structured interviews using seven open questions to be discussed. Based on the earlier interviews, some questions were added. All interviews were recorded after the participant was explained and confidentiality was established. First, they were asked to narrate the story of their lives after divorce. Then, during the interviews, the researcher tried to probe their narratives to explore how they coped with their problems and reregulated their lives. The researcher read the interviews immediately after transcription to grasp a sense of the whole. Afterward, the texts were divided into condensed meaning units, abstracted and labeled by codes. These codes, in turn, were compared in terms of similarities and differences and were integrated into categories and subcategories. Finally, the latent content was extracted into the main theme. Data gathering continued until data saturation was obtained.

### Data Analysis

According to the approach of Clarke and Braun, data analysis started after the fourth interview [11]. It began simultaneously with data gathering using the content analysis method. Immediately after an interview, the recorded data was transcribed and read line by line several times. Meaningful units of the transcripts (words, lines and paragraphs) were analyzed, and key concepts were taken as codes. Researchers summarized, grouped, and categorized the codes that emerged for concept similarity and contrast. This pattern of classification continued for all codes and categories until the emergence of subcategories and main categories. Two social workers who were not directly involved in the research carried out all the codifying process separately, and coding results were somehow modified considering the two other comments.

Meanwhile, the whole research team reviewed the emerging findings in their regular meetings, discussing tentative concepts and categories to reach a consensus about the results. After 13 interviews, the researcher provided the results to three experts, one sociologist, one psychologist, and one social worker who have considerable academic works and experience in this scope to verify the results and check if any area is not covered in the interviews. Finally, the latent contents of categories were formulated in the main themes.

### Data trustworthiness

To ensure the trustworthiness of the data, four criteria were used: credibility, conformability, dependability, and transferability. In this pursuit, prolonged engagement with participants helped attract their trust, allowing us to understand their experiences better and ultimately increased the credibility of the data. Member and external checking were used to secure the accuracy of the findings. Interviews, as well as emerging codes and categories, were continuously reviewed by the research team, and disagreements were solved through discussion. The strategy of maximum variance sampling and comparison of the results with previous studies were also applied.

## Results

Based upon the remarks of the participants, remarriage changes the relationship framework for both sides involved. Four categories on the main theme of inter-relationships that encompasses relationship with the spouse, with the family (reconfiguration of relationships with parents and in-laws), with children (impacts on rearing children and stepchildren), and with others (importance of not-important acquaintances) were found during this research (Table 1).

**Table 1.** Categories and subcategories of relationships with others.

Categories	Subcategories
**Relationship with the spouse**	1. Power balance
	2. Fragile emotions and regrets
	3. Coalition of convenience
**Reconfiguration of relationships with blood family and in-laws**	1. Totally disconnectedness or ceremonial connections
	2. Disturbed or conflictual relationships
	3. I am still the child of my family
**Impacts on rearing children and stepchildren**	1. Doubtful sense of parental love
	2. Early adulthood
	3. Looking for a reintegrated family
**Importance of unimportant acquaintances**	1. Significance of unimportant acquaintances
	2. Sympathetic friends
	3. Fear of contagion

### 1. Relationship with the spouse

As in the first marriage, spouses tend to react mostly against reflections between themselves in the remarriage process, and the partner is a salient pillar in all shared living. Hence, the biggest part of the interviews was designated to investigate the reciprocal behaviors of couples.

For the first category, three subcategories (power balance, fragile emotions, and coalitions of convenience) were extracted:

a) *Power balance:* To frame power directions inside the relationship, spouses may adopt different strategies. One or both sides perform passively and consequently excessive attention-seeking behaviors so that the couple relation transforms into a parent-like relationship.

b) *Fragile emotions and regrets:* As a common challenge, particularly in the first year of marriage, men or women are afraid of their partner's betrayal, especially for those couples who have divorced a second time or more. However, since the participants are still living together, it is interesting to listen to their ideas in this respect.

*“See, my husband's first wife had betrayed him and dated one of his friends, and then she went away. Although this made him feel suspicious, now we have been married for 30 years, and he is not suspicious of me although he used to be” (P-3, woman, 57-year-old).*

Some other participants pointed out that they cherished the ex-partner and blamed themselves for their insufficient attempts to keep the first love even after 10 or 30 years:

*“If I could be able to return to my previous life, I would act differently; it was my idiocy. In case it happens again, I will bottle up for six months, go to the best counselor, read books and ask to know what to do. My wife tells me that she shares the same feelings” (P-12, man, 37-year-old).*

Experts believe those who go through a so-called “hard divorce” (contested, unilateral, or multiple conflicts) are less likely to think of or regret memories. Of course, almost all acknowledged being committed to their current shared life.

c) *Coalition of convenience:* The patients were reluctant to disclose subtle discord with the person they are currently living with, letting alone marital problems that might be referred to as a deep conflict. They easily noted problems they had with children or family members, except the spouse. As agreement grew, interviewees explained that they normally avoided expressing their conflicts to remain safe from being blamed by others for the second mistake or guiltiness. Therefore, they adopted a strategy of hiding conflict and agreed to keep disagreements away from others.

### 2. Reconfiguration of relationships with families

In Eastern culture, the importance of in-laws is doubled because the kinship network is profoundly influential on marriage. For this category, three subcategories were identified:

a) *Totally disconnectedness or ceremonial connections:* This is a mechanism to minimize both consanguineous and affined interference of families in their dyad relationship to keep their privacy safe. The relationship in this part may vary from completely disconnected to present only in ceremonies with the least of sympathy. Most importantly, since spouses believed families played a destructive role in their previous marriage, they decided to cut down the relationships with families.

Sometimes, they even obviously show that they are glad for cutting the connection to the families. A young man who is experiencing his third marriage explained this cut-off with a sense of regret and fear:

*“We have no connection at all. We are rejected, but it does not matter very much. I count on myself since they tend to interfere even in the slightest affairs. They frequently interfered and damaged my former life. I prefer not to have any relationships” (P-8, man 37-year-old).*

b) *Disturbed or conflictual relationships:* Some families blame their child for the break-up of the first marriage. Although they still tend to maintain the relations, they frequently revenge on the newcomer by sarcastic comments. The most common scolds refer to comparing the new bride with the previous one or reproaching the son in front of his beloved for his faults.

c) *I am still the child of my family:* Some participants frankly stated that they were an inseparable member of the family and could not consider themselves apart from that, no matter their own or spouse's families. This is somehow in common for both first and next marriages. It appears to be normal within the families who consider the private life of any members within the bearing process.

### 3. Impacts on children rearing

Children deal with many changes, including new parents and step-siblings who do not know each other. Nevertheless, children's reactions are crucial in some ways for remarried couples.

a) *Doubtful sense of parental love:* some participants said they do not know how to behave properly with a newly arrived child since they never had a child. Moreover, some others described a sense of doubtfulness, particularly in the early years of remarriage. They commented that they tried not to express out, but frequently feel that when their spouse cared or played with children, s/he used a different way of contact towards the step-child. However, a worse situation was shaped when the child was living with the ex-partner and only sometimes goes to the recomposed family.

b) *Early adulthood:* some participants mentioned that their remarriage affected children to a large extent, pushing them to change and hurry to become adults. Thus, remarried parents are sometimes not unhappy if their child grows up sooner and leaves childish behaviors which could negatively influence dyad relations. That manner that annoys parents and leads them to warn others not to marry again unless children become adequately grown:

*“To me, if a second marriage is supposed to take place, it is better to postpone it to when children grow. With little kids, it is tough. I recommend others to wait before marrying again at least until the children are past the school-age (P-2, woman, 57 years old)”.*

c) *Looking for a reintegrated family:* if children refuse to accept their recomposed family as a real family, it will put parents into significant trouble. A sense of unitedness is more difficult to shape when the ex-father or mother is still in contact, or the child has to or is eager to see him/her frequently.

*“The only problem we have is that when my daughter goes to see her mother because some difficulties happen. She finds a dual feeling and comes back and says that her mom said this or that. I criticize her and tell her that this is your home and whatever she hears there should not be transferred here” (P-2, man 47 years old).*

Apparently, parents who let children be familiar with their upcoming step-siblings before they start their official marriage report a managed and well-functioned linkage.

### 4. Significance of unimportant acquaintances

This subcategory is capable of being upgraded to a category. Therefore, we should start with this relationship.

a) *Significance of unimportant acquaintances:* participants, especially those who had blood relativeness, described the situation by the expression of “we will clap eyes on each other”. In order to give up communication with the ex-in-laws, they stop or communicate less with all relatives. The same would occur for the shared friends and acquaintances of the previous shared life.

b) *Sympathetic friends:* Contrary to old friends, friendships made after the remarriage can provide a precious source of sympathy. However, having prior unpleasant experiences, remarried divorcees are reluctant to share their privacy, so friends can not help emotionally.

*“I do not like to share my personal problems with anybody. Of course, I made my decision to marry, but you know what, I want to live away from gossips. I fear losing my shared life. I fear turning back to the first point as I used to be. These fears cause me to stay away from people” (P-4, man, 37 years old).*

The fear of stories is not only for remarried persons, but people around them say the same about the man or woman once divorced.

c) *Fear of contagion:* After remarriage, the stigma of divorce will not be completely removed. For this reason, many remarried persons (of both genders) strive not to be recognized and, in many cases, move to new houses to avoid being judged by others. Two conducted interviews are suggestive of this:

*“When they see newly married couples, neighbors look freakily at them and try to keep husbands away from brides. They think it is contagious. It is awful that this will not change till the end of life, and it makes one cut the relationship (P-14, woman, 36 years old)”.*

*“My last word to him was that his mother and sisters should never call me a divorcee. I hated this word. I have adult children, and I am afraid of what somebody who would want an engagement with them would think if they realize their mom is divorced. I told him that once he or his family said that word, I would immediately terminate the relationship with him” (P-2, woman, 57 years old).*

## Discussion

In this study, lived experiences of persons who remarried after a divorce was investigated. A content analysis was employed to grasp the experiences. Due to the stigma of divorce, many candidates refused to take part, but eventually, 17 individuals – 8 women and 9 men shared their experiences with the team. In all the forms of relations, it was important for researchers to check the behavior differences in the first and second marriages to make sure they are affected by the occurrence of remarriage after divorce.

For the “relationships with others” theme, four categories were discovered: “relationships with spouse”, “reconfiguration of relationships with families”, “impacts on child-rearing”, and “significance of unimportant acquaintances”, each with different subcategories.

In a conjugal relation, it is critical to redefine trade-off; different points are found in the second marriage compared to the first one because, in the second relationship, it is expected that both sides avoid repetition of mistakes of the previous shared life and launch the relation more cautiously and maturely. In conformity with previous studies, by increasing age at the time of marriage, particularly for women, they attempt alertly to discuss their status in the marriage with respect to decision-making on childbearing, financial affairs, and personal matters like job, education and relations with their own families [12, 13].

On the other hand, since marriage is essentially assumed as an emotional contract, it is not unusual that couples believe that an emotional relationship is important. It should be noted that emotions could be fragile in remarriage after divorce due to what the individuals think about the inherent essence of this phenomenon. According to Garneau *et al.*, emotions in a second marriage following a divorce are built on the way the spouses view their new condition [14]. The finding complies with previous studies that remarriage is largely under the influence of the previous marriage [4, 15, 16]. Actually, remarried persons have a history of divorce that is not pro-social. Hence, it may deeply hurt their emotions and trust. Also, the regret of lost life and distrustfulness to new partner became highlighted in this study, in accordance with Pirak *et al.* [8], Knox *et al.* [15], and Zare *et al.* [7], showing that fear of repetition of bad outcomes stands along with remarriage, at least in the beginning years. It can be a reason for what we called a “coalition of convenience”. This term is hired from politics but conveniently suits the situation in which two sides agree to build and show a normal facet to others, especially significant others, about their disputes and conflicts, a behavior that is less common in the first marriage. They hide their conflicts even from their families to avoid being judged and blamed. In the report of Lee *et al.*, family and marriage are much respected with an eastern cultural view, and their underestimation by a liberal or negligent behavior may be strongly reacted by the context [17].

According to the studies from other Asian societies, in the Iranian context, even after the collapse of the first marriage, a family must be a vital point for anyone who intends to work in the remarriage field [18]. Lee *et al.* stated that parents entitle themselves to meddle in their children's lives in Korean society because they assume they have invested their time and money in bearing children. Hence, they have the right to make sure that these resources are not easily lost [17]. Our findings reaffirmed those of Read *et al.*, who stated that spouses are not the only persons who are connected by remarriage, but they are just as essential atoms for a large kinship network in which children are also included [19].

Children are always important in the relationships between spouses. However, when marriage after divorce is considered, situations change. For example, step-children are potentially an excuse for the parents to recognize each other's goodwill and possibly a degree of sacrifice. The results also stress that the age of children can make a difference in the quality of relationships between couples. Shrifter *et al.* found out that new couples are likely to experience satisfaction from their new shared life when children are younger at the beginning of remarriage [20]. Otherwise, there is evidence to approve that difficulties in caring for babies can put couples into trouble.

An optimistic point was seen in interviews showing that children are likely to adapt to the family transition, contrary to the findings of Pagani *et al.*[21] and Warsha *et al.* [22]. Moreover, Shrifter *et al.* [20] and Hetherington *et al.* [23] indicate that individual characteristics for communicative and confrontation abilities support this finding, as suggested by Greeff *et al.* [24] and Hetherington *et al.* [25].

In accordance with what Higgins *et al.* asserted, friends can have a positive impression by giving emotional support during the period between two marriages and the first period of remarriage [26]. Also, a continuation of linkage with social network components in common with an ex-partner or ex-in-law family (when they were relatives or still live in the same neighborhood) may negatively sensitize new spouses. That is more important for cases where the divorce was uncontested or agreed upon since, as mentioned, doubt to betray is of tremendous risk for remarriage, which occurs following a not-hard divorce [27]. Therefore, by custodial and financial affairs, the ghost of far related persons may still affect the quality of marital relationships after remarriage. The last notion involves a context-oriented behavior that the divorcee is never presumed as innocent even if s/he comes to a new tie. This manner is mostly reflected in neighbors, and it is a concern for female remarried divorcees that women in the community have exceeded fear from the person who has had an experience of divorce and try to prevent their husbands from getting close to them even in a social association. Challenges continue even when children of the new marriage are born [28]. Some studies highlighted the results of the present study for the blended families and showed the effects of social and cultural context on remarriage, particularly after divorce [28–30]. To implicate the media in the discussion, Higgins *et al.* argue that disapproving public view is not still in place because underlying causes like the media are strongly involved [26]. Nonetheless, we think that an important limitation of this study was that many target subjects hardly agreed to start an interview and preferred to fill up questionnaires rather than speak about their “bitter past”.

## Conclusion

This study revealed the complexity of the relationship network when another marriage happens after the first one collapsed. Another result of this study was that relations after divorce might not disappear completely, and relics of the first ruined shared life may strongly affect the new partnership. In this research, the researchers focused on the social life that impacts the relationship between the two sides of remarriage. Notwithstanding, future studies should take notice of other aspects of remarriage-related connections, including the impacts remarriage may have on childbearing or the relations of two families with each other.

The results of this study are helpful for different groups. Scholars who are engaged with the study of divorced and remarried persons have a piece of evidence for future investigations. Practitioners of helping professions can use the results of the study in planning and applying helpful plans and consultations. Policymakers are key players who should know that remarriage, albeit all challenges, could have a positive and improving impact on the lives of the divorced men and women; they may consider ways to support the second marriage for the families who were not successful, irrespective of the reason.

## Acknowledgments

This study was part of Abbasali Yazdani's PhD thesis at the University of Social Welfare and Rehabilitation Sciences.

### Ethical approval

The approval for this study was obtained from the Ethics Committee of the University of Social Welfare and Rehabilitation Sciences (approval ID: IR.USWR. REC.1396.313).

### Consent to participate

All participants were called before arranging a meeting, and all knew about the procedure and interview goals. In addition, oral consent for participation and recording of the interviews was taken. They were reassured of the anonymity and confidentiality of their identity. The voluntary nature of their participation was emphasized, and they were explained that they could discontinue participating or withdraw from the study whenever they wanted.

### Conflict of interest

The authors declare that there is no conflict of interest.
